# Immunological Insights into Peritoneal Carcinomatosis for Gastrointestinal Malignancies: The Role of Soluble Factors in Malignant Ascites

**DOI:** 10.3390/biomedicines14051141

**Published:** 2026-05-18

**Authors:** Ufuk Oguz Idiz, Ilhan Mutlu, Yucel Barut, Eyup Kaya, Aysegul Ferlengez, Ihsan Gunduz, Taskin Rakici, Erdem Kinaci, Mahmut Emin Cicek, Anil Demir, Musa Murat Caliskan, Murat Altinkaynak, Erol Aydin, Mert Ali Dolek, Selim Dogan, Yurdakul Deniz Firat, Mert Mahsuni Sevinc

**Affiliations:** 1 Department of General Surgery, Istanbul Training and Research Hospital, 34098 Istanbul, Turkey; 2 Department of Radiology, Basaksehir Cam and Sakura City Hospital, 34480 Istanbul, Turkey; 3 Department of Radiology, Prof. Dr. Cemil Tascioglu City Hospital, 34384 Istanbul, Turkey; 4 Department of Anesthesiology and Reanimation, Istanbul Training and Research Hospital, 34098 Istanbul, Turkey; 5 Department of General Surgery, Tekirdag Dr. Ismail Fehmi Cumalioglu City Hospital, 59030 Tekirdag, Turkey; 6 Department of Radiology, Istanbul Training and Research Hospital, 34098 Istanbul, Turkey; 7 Department of General Surgery, Basaksehir Cam and Sakura City Hospital, 34480 Istanbul, Turkey; 8 Department of General Surgery, Bursa Yuksek Ihtisas Training and Research Hospital, 16310 Bursa, Turkey

**Keywords:** gastric cancer, colorectal cancer, pancreatic cancer, immune checkpoints, cytokine

## Abstract

**Background and Objectives:** Malignant ascites reflects the tumor microenvironment and provides valuable insights into peritoneal metastasis. This study aimed to assess soluble immune system-related molecules in the ascites fluid of advanced gastrointestinal cancer patients with peritoneal carcinomatosis and to explore potential therapeutic opportunities. **Methods:** This multicenter prospective cohort study included 48 patients with gastrointestinal adenocarcinoma (17 colorectal, 16 gastric, and 15 pancreatic) with malignant ascites and 15 patients for comparison who required benign ascites drainage for advanced heart failure. Blood samples for routine parameters and ascites fluid for cytokine and soluble immune checkpoint analysis were collected. Parameters were compared between cancer patients and the comparison group, across cancer subgroups, and in correlation with survival. **Results:** The mean age of participants was 60.2 ± 14.9 years, with a female-to-male ratio of 11:20. The median survival of cancer patients was 84.0 days. Compared with heart failure-associated transudative ascites, malignant ascites demonstrated higher levels of TNF-α, IL-6, IL-10, IL-12p70, IL-18, IL-23, s4-1BB, and TGF-β1, while albumin levels were lower. Significant intergroup differences were observed in TNF-α, IL-6, IL-8, IL-10, IL-12p70, IL-23, 4-1BB, TGF-β1, and PD-L1 levels. In exploratory multivariable analysis, IL-10 and soluble 4-1BB emerged as potential predictors of shorter survival, although these findings require validation in larger cohorts. Survival was negatively correlated with PD-L1, TNF-α, IL-6, and IL-10 in colorectal cancer; 4-1BB, TGF-β1, IL-8, and IL-10 in gastric cancer; and TGF-β1, IL-6, and IL-10 in pancreatic cancer. **Conclusions:** These exploratory findings indicate that the immunosuppressive milieu in malignant ascites in gastrointestinal cancers may be mediated by cancer subtype-specific pathways, warranting further mechanistic and translational investigation.

## 1. Introduction

Gastric, colorectal, and pancreatic cancers are common gastrointestinal malignancies that often progress to advanced stages with poor prognoses. Malignant ascites develops due to peritoneal cancer spread, with only 11% of patients surviving beyond six months [1,2]. It contains cancer cells, immune cells, cytokines (e.g., VEGF-A, IL-10, TGF-β1), and immune checkpoint molecules that collectively shape an immunosuppressive microenvironment [3,4].

Ascites fluid acts as a liquid biopsy of the peritoneal cavity, capturing the aggregate secretory output of the entire peritoneal tumor burden rather than a single-lesion snapshot and reflecting locally concentrated rather than systemically diluted immune signals [5]. Although extensively studied in ovarian cancer, where cytokine and checkpoint profiles influence prognosis and drug resistance [6,7], data on the immune composition of gastrointestinal cancer ascites remain limited.

Immune checkpoint inhibitors (ICIs) have transformed cancer immunotherapy by enhancing antitumor immunity, yet resistance remains a major challenge, and reliable response biomarkers are still needed [8]. Soluble immune checkpoints, generated by proteolytic cleavage or alternative mRNA splicing, are thought to bind the same ligands as their membrane-bound counterparts and may modulate cytokine secretion, T-cell survival, and T-cell proliferation, making them candidate prognostic indicators [9,10,11].

Despite these advances, the immune role of ascitic fluid in peritoneal carcinomatosis remains underexplored. Therefore, we investigated soluble immune mediators in ascites from patients with advanced gastrointestinal cancer, with the goal of identifying potential therapeutic targets.

## 2. Materials and Methods

This study was designed as a multicenter prospective cohort study and commenced following approval from the local clinical research ethics committee. Written informed consent was obtained from all participants, and the study was registered in a public trials system (NCT04566848).

### 2.1. Patients

**Figure 1:**
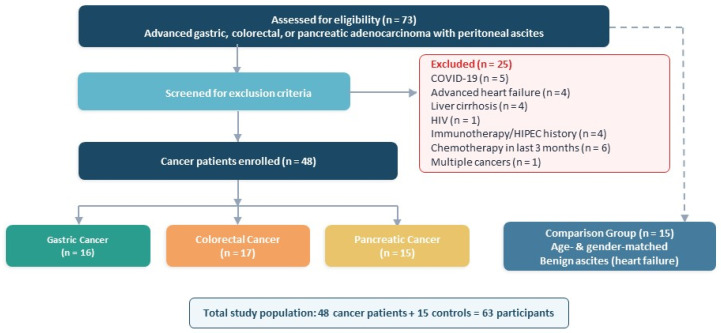
Flow chart shows the status of gastrointestinal cancer and comparison group included in the study according to exclusion criteria.

The study included 73 volunteers with advanced gastric, colorectal, or pancreatic adenocarcinoma and intra-abdominal ascites due to peritoneal carcinomatosis, who visited the general surgery clinic and the radiology department between 1 October 2020 and 1 January 2022. Based on the exclusion criteria, 48 cancer patients were selected consecutively, excluding those with COVID-19, advanced heart failure, liver cirrhosis, HIV, pregnancy, a history of immunotherapy or HIPEC, chemotherapy within the last 3 months, or multiple cancers. Although prior surgical status related to the primary cancer and the chemotherapy regimen received were not considered to be exclusion criteria, given the current metastatic status of the disease, detailed clinicopathological characteristics, including tumor stage, prior treatments, histological subtype, and liver metastasis status, were noted. Patients were categorized into gastric, colorectal, and pancreatic cancer groups. Additionally, fifteen age- and gender-matched volunteers requiring benign ascites drainage due to advanced heart failure were included as a comparison group based on hospital admission order. Advanced heart failure patients were selected as the comparison group because (i) they presented with clinically significant, non-malignant ascites requiring drainage, thereby providing a procedurally matched sampling condition, (ii) heart failure-related ascites is predominantly a transudative, mechanically driven process with minimal local inflammatory or immunological activation, offering a relatively immunologically inert baseline against which cancer-driven immune perturbations could be highlighted, and (iii) cirrhotic patients were deliberately excluded because cirrhosis itself induces substantial peritoneal immune activation, elevated cytokine levels (IL-6, TNF-α, TGF-β1), and bacterial translocation-driven inflammation, which would have confounded the interpretation of cancer-specific immune signatures rather than clarifying them (Figure 1).

### 2.2. Parameters and Measurement

Age, gender, complete blood count, INR, and biochemical markers were assessed in all volunteers before ascites sampling. A 10-cc ascites sample was collected via a drainage catheter inserted in the left lower quadrant under ultrasound guidance or direct vision. Samples were centrifuged at 1800 rpm for 10 min to separate the serum, which was stored at −80 °C in labeled 2 mL Eppendorf tubes for cytokine and immune checkpoint analysis.

Once all samples were collected, they were thawed for measurement. Cytokines (IL-1β, IFN-α2, IFN-γ, TNF-α, MCP-1, IL-6, IL-8, IL-10, IL-12p70, IL-17A, IL-18, IL-23, IL-33) were analyzed using the LEGENDplex™ Human Inflammation Panel 1 (Biolegend, San Diego, CA, USA, cat no: 740808). Soluble immune checkpoints (CD25, 4-1BB, CD86, Free Active TGF-β1, CTLA-4, PD-L1, PD-1, Tim-3, LAG-3, Galectin-9) were measured with the LEGENDplex™ HU Immune Checkpoint Panel 1 (Biolegend, San Diego, CA, USA, cat no: 740961) using flow cytometry (Cube 8™, Sysmex, Kobe, Japan, cat no: CY-S-3068R_V3). Cytokine measurements were performed as single determinations per sample, while soluble immune checkpoint analyses were performed in duplicate, with mean values used for statistical analysis. This approach was adopted due to limited ascites sample volume available per patient and the cost constraints of the bead-based multiplex platform. The LEGENDplex™ (Biolegend, San Diego, CA, USA, cat no: 740808) assay employs an internal standard curve with known concentrations run alongside samples; values falling below the lower limit of quantification (LLOQ) of the standard curve were recorded as such. For analytes where a substantial proportion of measured values fell near or below the assay’s minimum detectable concentration (MDC)—including TNF-α, IL-8, and IL-23—results should be interpreted with caution, as measurement precision is inherently reduced at the lower boundary of the dynamic range.

### 2.3. Volunteers Grouping for Statistical Analysis

Statistical analysis was conducted in two stages. First, all cancer patients were grouped together and compared with the comparison group. Second, cancer patients were divided into gastric, colorectal, and pancreatic subgroups, and all parameters were compared across these four groups.

### 2.4. Statistical Analysis

Data were analyzed using SPSS 26.0, R-Studio Build 446, and GraphPad Prism V.9.3.1. Normality was assessed with the Kolmogorov–Smirnov test, and variance homogeneity with Levene’s test. Student’s t-test was used for normally distributed data, while the Mann–Whitney U test was applied for non-normally distributed data. One-way ANOVA and Kruskal–Wallis tests were used for multiple-group comparisons, followed by the Bonferroni test for post hoc analysis. Qualitative variables were analyzed with the Chi-square and Fisher’s exact test. We constructed Kaplan–Meier curves by cancer subtype and reported median survival with 95% confidence intervals. Group differences were assessed using the log-rank (Mantel–Cox) test. Univariable and multivariable Cox proportional hazards regression analyses were performed to evaluate independent predictors of decreased survival. Pearson correlation coefficients were used to assess survival correlations, with the following interpretations: 0.00–0.19 (very weak), 0.20–0.39 (weak), 0.40–0.59 (moderate), 0.60–0.79 (strong), and 0.80–1.0 (very strong). Given the limited sample size and the exploratory nature of the study, the multivariable Cox model should be interpreted with caution. With 48 events, the commonly cited guideline of approximately 10–15 events per predictor variable limits the number of covariates that can be reliably estimated. Variables included in the multivariable model were selected based on univariable significance (*p* < 0.05); however, we acknowledge that this data-driven selection approach in a small cohort increases the risk of overfitting. No formal collinearity diagnostics (e.g., variance inflation factors) or internal validation procedures (e.g., bootstrapping, cross-validation) were performed; therefore, the reported hazard ratios should be considered preliminary estimates requiring validation in larger cohorts. To assess whether systemic clinical parameter imbalances confounded the ascites immune mediator profiles, Spearman rank correlation analyses were performed between key clinical variables (albumin, AST, direct bilirubin, and platelet count) and each significantly altered ascites biomarker, both in the pooled cancer cohort and stratified by cancer subtype.

## 3. Results

This study included 48 patients with gastrointestinal adenocarcinoma (17 colorectal, 16 gastric, and 15 pancreatic) and 15 volunteers with benign ascites for comparison. The mean age was 60.2 ± 14.9 years, with a female-to-male ratio of 11/20. All patients died during follow-up after study enrollment; the maximum observed survival was 156 days. The overall median survival for cancer patients was 84.0 (95% CI 80.6–87.3) days, varying by subtype: 88.0 (95% CI 79.9–96.0) days for colorectal, 88.0 (95% CI 80.1–95.8) days for gastric, and 72.0 (95% CI 62.5–81.4) days for pancreatic cancer. Demographic data, blood counts, and biochemical parameters are shown in Supplementary Table S1. Neutrophil counts, AST, and ALT levels were significantly higher in cancer patients (*p* = 0.032, *p* = 0.016, and *p* = 0.029, respectively), while albumin levels were lower (*p* = 0.012).

Given the transudative nature of heart failure-related ascites versus the exudative and biologically active nature of malignant ascites, between-group differences should be interpreted as reflecting both disease-specific immune mechanisms and inherent differences in ascites pathophysiology. To contextualize these findings, we emphasize that intergroup comparisons among cancer subtypes—which share a common exudative ascites biology—may provide more reliable insights into tumor-specific immune variation than comparisons with the heart failure group.

The clinicopathological characteristics of the 48 cancer patients are detailed in Table 1. All patients had Stage IV disease with peritoneal carcinomatosis at enrollment. The majority of tumors were poorly differentiated (60.4%), consistent with the advanced disease stage. Prior tumor resection had been performed in 33 patients (68.8%), with notably lower resection rates in the pancreatic subgroup (46.7%) than in the colorectal (82.4%) and gastric (75.0%) groups. Liver metastases were present in 26 patients (54.2%), with comparable distribution across subgroups (Table 1).

**Figure 2:**
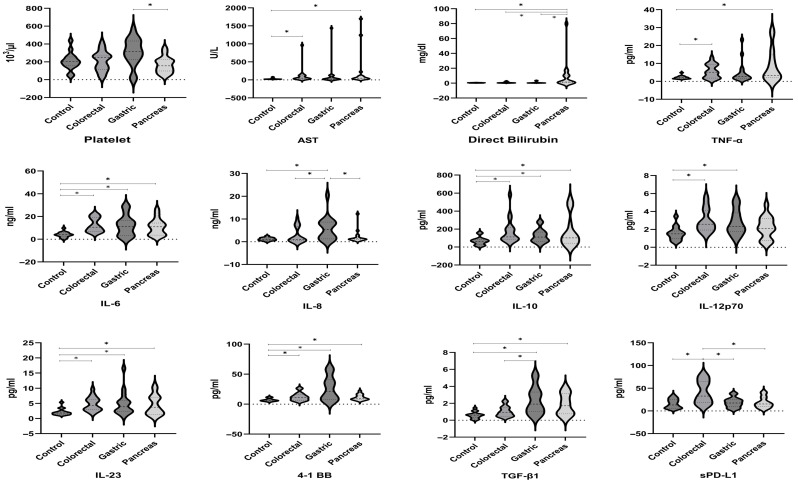
Distribution of ascites-fluid biomarkers and clinical parameters across study groups. Multi-panel plot showing the variables that differed significantly among the comparison group and cancer subtypes. Panels include cytokines and soluble immune checkpoints with between-group differences (TNF-α, IL-6, IL-8, IL-10, IL-12p70, IL-23, s4-1BB, TGF-β1, sPD-L1) and selected laboratory parameters (platelets, AST, direct bilirubin). Distributions are displayed as box-and-whisker plots (center line = median; box = IQR; whiskers = range; points = outliers). Omnibus comparisons were performed using one-way ANOVA (platelet) with Bonferroni post hoc tests and Kruskal–Wallis (for the other parameters) as appropriate with pairwise contrasts; significance is annotated in the panels (* *p* < 0.05). Measurements are from ascitic fluid collected at enrollment (units as labeled in each panel).

Cytokine and soluble immune checkpoint levels in ascitic fluid were significantly elevated in cancer patients, including TNF-α, IL-6, IL-10, IL-12p70, IL-18, IL-23, s4-1BB, and TGF-β1 (*p*-values < 0.05, Table 1). Cytokine levels in ascitic fluid varied by cancer type. TNF-α, IL-6, IL-8, IL-10, IL-12p70, and IL-23 were significantly different between cancer subtypes and the comparison group. Notable differences included TNF-α (*p* < 0.05, colorectal/pancreatic vs. comparison group), IL-6 (*p* < 0.05, all cancer subtypes vs. comparison group), IL-8 (*p* < 0.05, gastric vs. other groups), IL-10 (*p* < 0.05, all cancer subtypes vs. comparison group), and IL-23 (*p* < 0.05, all cancer subtypes vs. comparison group), IL-12p70 (*p* < 0.05, comparison group vs. colorectal/gastric), IL-23 (*p* < 0.05, all cancer subtypes vs. comparison group) (*p* < 0.05, Table 2, Figure 2).

Significant differences in platelet counts, AST, and bilirubin levels were observed among gastrointestinal cancer subgroups and the comparison group. Platelet counts differed between gastric and pancreatic cancer (*p* = 0.006); AST levels differed between the comparison group and pancreatic (*p* = 0.012) and colorectal cancer (*p* = 0.018), and direct bilirubin levels differed between pancreatic and colorectal, gastric, and the comparison groups (*p* < 0.05). Albumin levels were significantly lower in pancreatic cancer than in the comparison group (*p* = 0.040, Supplementary Table S2, Figure 2).

Notably, the pancreatic cancer subgroup exhibited significantly higher direct bilirubin and AST levels and lower albumin levels compared with other groups, reflecting the obstructive biliary pathology and hepatic involvement characteristic of advanced pancreatic malignancy. Similarly, the gastric cancer group demonstrated higher platelet counts, possibly related to chronic inflammatory thrombocytosis. These systemic parameter imbalances represent potential confounders in interpreting ascites cytokine and immune checkpoint profiles, as hepatobiliary dysfunction, systemic inflammation, and hypoalbuminemia may independently influence peritoneal immune mediator concentrations.

Spearman correlation analyses between these systemic parameters and ascites immune mediators revealed minimal confounding: only 4 of 144 tested correlations were significant, with none involving the primary biomarkers identified in survival analyses (TNF-α, IL-6, s4-1BB, TGF-β1). The only notable association was a weak negative correlation between albumin and IL-10 in the pooled cancer cohort (ρ = −0.307, *p* = 0.034), suggesting that the observed subtype-specific immune profiles are predominantly driven by tumor biology rather than systemic parameter imbalances (Supplementary Table S3).

Furthermore, immune checkpoint levels in ascites fluid varied by cancer type. Free Active TGF-β1, 4-1BB, and sPD-L1 were significantly different between cancer subtypes and the comparison group. s4-1BB (all cancer subtypes vs. comparison group, *p* < 0.05), TGF-β1 (gastric vs. colorectal and comparison group vs. pancreatic/gastric, *p* < 0.05), and sPD-L1 (colorectal vs. other groups, *p* < 0.05) showed significant differences (Table 3, Figure 2).

Kaplan–Meier curves with log-rank (Mantel–Cox) tests demonstrated significant differences in survival across cancer subgroups, with pancreatic cancer exhibiting the shortest median survival (*p* = 0.016). In an exploratory multivariable Cox model, IL-10 (HR = 1.007, 95% CI 1.002–1.012, *p* = 0.007) and soluble 4-1BB (HR = 1.034, 95% CI 1.002–1.067, *p* = 0.036) were retained as potential predictors of shorter survival. However, given the limited sample size (n = 48, 48 events), the number of candidate covariates relative to events raises concerns about model overfitting, and these results should be regarded as hypothesis-generating rather than confirmatory. Furthermore, the high degree of intercorrelation among inflammatory mediators (as reflected in the correlation heatmaps, Supplementary Figure S2) introduces potential collinearity, which may affect coefficient stability and inflate or attenuate individual hazard ratios (Table 4).

The correlation heatmaps of all parameters evaluated with survival in gastrointestinal cancer patients, stratified by GI cancer subtype, are shown in Supplementary Figure S2. Significant negative correlations were observed in colorectal cancer between sPD-L1 (*p* = 0.019, r = −0.560), TNF-α (*p* = 0.001, r = −0.744), IL-6 (*p* = 0.035, r = −0.514), and IL-10 (*p* = 0.013, r = −0.588). In gastric cancer, significant negative correlations were identified for s4-1BB (*p* = 0.029, r = −0.544), TGF-β1 (*p* = 0.019, r = −0.578), IL-8 (*p* = 0.032, r = −0.536), and IL-10 (*p* = 0.003, r = −0.700). In pancreatic cancer, significant negative correlations were observed for TGF-β1 (*p* = 0.043, r = −0.529), IL-6 (*p* = 0.015, r = −0.615), and IL-10 (*p* = 0.007, r = −0.664), as shown in Supplementary Figure S2.

## 4. Discussion

This study presents a comprehensive analysis of soluble immune mediators in malignant ascites from advanced gastrointestinal cancers. Cytokines such as TNF-α, IL-6, IL-10, and TGF-β1, together with the soluble immune checkpoints 4-1BB and PD-L1, were significantly elevated in peritoneal fluid, and their profiles varied by cancer subtype. These findings indicate that malignant ascites harbors an immunosuppressive milieu that may facilitate tumor immune evasion through subtype-specific mechanisms [7,12].

Unlike serum biomarkers, ascites fluid concentrates locally produced immune mediators and more directly reflects the peritoneal tumor microenvironment. Furthermore, unlike tumor tissue biopsies, ascites fluid integrates secretory products from the entire peritoneal tumor burden, potentially offering a more representative picture of the overall immune landscape in disseminated disease. This rationale is consistent with prior ovarian cancer studies showing that ascites-derived immune profiles differ from matched blood and tissue analyses and may carry independent prognostic value [6,7].

Pro-inflammatory cytokines—IL-6, TNF-α, IL-8, IL-12p70, and IL-23—were broadly elevated in our cohort and align with their established roles in shaping a tumor-permissive immune environment. IL-6 promotes immune evasion by driving immunosuppressive activity and macrophage skewing, while TNF-α enhances tumor adhesion, growth, and metastasis [5,13,14]. Both were elevated across all gastrointestinal cancers, particularly in colorectal and pancreatic tumors, and correlated with poorer survival. IL-8, a chemokine driving neutrophil recruitment and angiogenesis, was disproportionately elevated in gastric cancer—consistent with its association with Helicobacter pylori-driven inflammation, angiogenesis, and metastasis [15]—and correlated with shorter survival; to our knowledge, IL-8 levels in gastrointestinal cancer ascites have not been previously reported. IL-23, which promotes tumor growth via Th17-mediated inflammation [13,16,17], was highest in colorectal cancer, possibly reflecting microbiota-driven amplification. In contrast, IL-12p70, which drives Th1/IFN-γ-mediated antitumor immunity [18,19,20], was elevated mainly in colorectal and gastric cancers, likely representing an attempted but blunted antitumor response in the face of dominant immunosuppressive signaling.

Among immunosuppressive cytokines, IL-10 and TGF-β1 were both elevated in malignant ascites and consistently associated with shorter survival across all cancer subtypes. IL-10 directly suppresses T-cell function and supports immune evasion in gastrointestinal cancers [21], while TGF-β1—previously reported to be elevated in gastric cancer ascites compared with cirrhotic ascites [5]—synergizes with IL-10 to further inhibit effector T-cell activity [22]. Together, these findings support a coordinated immunosuppressive cytokine axis as a key driver of disease progression.

Soluble immune checkpoint proteins, generated through membrane cleavage or alternative splicing, have been linked to advanced stage, poor survival, and reduced treatment response across multiple cancers [23,24]. Soluble PD-L1 retains the inhibitory activity of its membrane-bound form by engaging PD-1 [25,26], and was significantly elevated specifically in colorectal cancer ascites—a finding not previously reported in gastrointestinal cancers, but consistent with ovarian cancer data showing higher peritoneal sPD-L1 levels and poorer survival in patients with elevated values [26]. Soluble 4-1BB (s4-1BB), generated by alternative splicing in activated T cells [27], can disrupt 4-1BB/4-1BBL co-stimulation and dampen effector T-cell responses [28]. Although prior reports have been mixed [23,29,30], we observed significantly elevated s4-1BB across all gastrointestinal cancer subtypes, with an independent association with shorter survival, suggesting that s4-1BB shedding contributes to the immunosuppressive milieu of malignant ascites.

The interpretation of ascites immune profiles in the pancreatic subgroup warrants additional pathophysiological consideration. Unlike colorectal and gastric cancers, where peritoneal carcinomatosis is predominantly driven by direct transcelomic shedding, pancreatic cancer-associated ascites may arise through multiple overlapping mechanisms, including peritoneal carcinomatosis, lymphatic obstruction due to lymphangiosis carcinomatosa, portal hypertension from direct tumor invasion or compression of the portal venous system, and hypoalbuminemia secondary to hepatobiliary dysfunction [31,32]. Lymphangiosis carcinomatosa—defined by tumor emboli within dilated lymphatic vessels—disrupts normal peritoneal lymphatic drainage and may produce chylous or protein-rich ascites with a distinct immunological composition compared to purely carcinomatosis-driven exudative ascites [31]. This mechanistic heterogeneity may partly explain the distinct cytokine and checkpoint profile observed in the pancreatic subgroup, including the prominent hepatobiliary-related confounders (elevated bilirubin and AST) discussed above. Consequently, the immune mediator levels measured in pancreatic cancer ascites likely reflect a composite of tumor-driven immune modulation, biliary obstruction-related hepatic inflammation, and lymphatic disruption, and these contributions cannot be individually quantified in the current dataset.

An important consideration when interpreting cancer subtype-specific immune profiles is the concurrent heterogeneity in systemic clinical parameters. The pancreatic subgroup, in particular, exhibited pronounced hepatobiliary dysfunction (elevated AST and direct bilirubin) and lower albumin levels, which may influence ascites composition through several mechanisms: (i) biliary obstruction can trigger hepatic acute-phase responses that alter peritoneal cytokine production; (ii) hypoalbuminemia increases capillary permeability and may alter the transperitoneal flux of soluble mediators; and (iii) cholestasis-associated immune dysregulation has been independently linked to elevated IL-6 and TNF-α levels [33]. Similarly, the higher platelet counts in the gastric subgroup may reflect chronic inflammatory thrombocytosis, with platelets themselves serving as sources of TGF-β1 and other immune mediators [34]. Reassuringly, systematic Spearman correlation analyses demonstrated negligible associations between these systemic parameters and ascites immune mediator concentrations, supporting the interpretation that the subtype-specific cytokine and checkpoint profiles reflect tumor-driven peritoneal immune modulation rather than confounding by hepatobiliary dysfunction or inflammatory thrombocytosis.

Several limitations should be acknowledged. First, the relatively small sample size limits the power of subgroup analyses and the reliability of multivariable Cox regression modeling; accordingly, the survival associations identified herein should be regarded as exploratory and require validation in larger cohorts. Second, cytokine measurements were performed as single determinations, which may reduce analytical precision near the lower detection threshold, although the consistency of our findings with published data supports their biological plausibility. Third, heart failure-associated transudative ascites was chosen as the comparator to provide an immunologically neutral baseline; while this limits generalizability to other non-malignant etiologies, it avoids the cytokine confounders inherent to cirrhotic ascites. Fourth, the absence of paired serum measurements, tumor tissue immunohistochemistry, and membrane-bound checkpoint expression analysis precludes compartmental gradient assessment and identification of the cellular sources of the soluble markers detected. Fifth, the clinical heterogeneity across cancer subgroups—particularly hepatobiliary dysfunction in the pancreatic subgroup and multifactorial ascites etiology including lymphangiosis carcinomatosa—represents a potential confounder, although correlation analyses demonstrated minimal impact on the primary findings. Future multicompartment studies with larger, treatment-stratified cohorts would address these limitations.

In conclusion, the elevated levels of specific cytokines (TNF-α, IL-6, IL-8, IL-10, IL-23, TGF-β1) and immune checkpoints (4-1BB, sPD-L1) in the ascites fluid of gastrointestinal cancer patients indicate a profoundly immunosuppressive peritoneal microenvironment. This environment likely plays a critical role in tumor immune evasion, with underlying pathways varying distinctly by cancer subtype. While our observational data do not establish direct causality, these subtype-specific immune profiles provide a strong foundation for future translational research. Specifically, they raise the hypothesis that targeting these pathways—potentially through locoregional immunotherapies—could favorably reprogram the peritoneal immune milieu. To translate these findings into therapeutic applications, further mechanistic studies, preclinical functional validations, and early-phase clinical trials are essential.

## Figures and Tables

**Table 1:** Clinicopathological Characteristics of Cancer Patients (NA: Data Not Avaible).

Characteristic	Colorectal (n = 17)	Gastric (n = 16)	Pancreatic (n = 15)	All Cancer (n = 48)
n	17	16	15	48
Age, years (mean ± SD)	61.0 ± 12.6	53.2 ± 16.4	63.2 ± 13.0	59.1 ± 14.5
Prior tumor resection n (%)	14 (82.4%)	12 (75.0%)	7 (46.7%)	33 (68.8%)
Stage IV	17 (100.0%)	16 (100.0%)	15 (100.0%)	48 (100.0%)
**Gender, n (%)**				
Male	12 (70.6%)	11 (68.8%)	9 (60.0%)	32 (66.7%)
Female	5 (29.4%)	5 (31.2%)	6 (40.0%)	16 (33.3%)
**Histological subtype, n (%)**				
Adenocarcinoma (NOS)	16 (94.1%)	13 (81.2%)	-	29 (60.4%)
Mucinous adenocarcinoma	1 (5.9%)	-	-	1 (2.1%)
Signet ring cell carcinoma	-	3 (18.8%)	-	3 (6.2%)
Ductal adenocarcinoma	-	-	15 (100.0%)	15 (31.2%)
**Tumor differentiation, n (%)**				
Poorly differentiated	10 (58.8%)	11 (68.8%)	8 (53.3%)	29 (60.4%)
Moderately differentiated	5 (29.4%)	4 (25.0%)	6 (40.0%)	15 (31.2%)
Well differentiated	2 (11.8%)	1 (6.2%)	1 (6.7%)	4 (8.3%)
**Last chemotherapy regimen, n (%)**				
FOLFOX	8 (47.1%)	12 (75.0%)	-	20 (41.7%)
FOLFIRI	2 (11.7%)	-	-	2 (4.2%)
5-FU	4 (23.5%)	-	-	4 (8.3%)
FLOT	-	2 (12.5%)	-	2 (4.2%)
FOLFIRINOX	-	-	6 (40.0%)	6 (12.5%)
Gemcitabine	-	-	7 (46.7%)	7 (14.6%)
NA	3 (17.6%)	2 (12.5%)	2 (13.3%)	7 (14.6%)
**Liver metastasis, n (%)**				
Liver metastasis present	10 (58.8%)	9 (56.2%)	7 (46.7%)	26 (54.2%)
None	7 (41.2%)	7 (43.8%)	8 (53.3%)	22 (45.8%)
<3	5 (29.4%)	8 (50.0%)	4 (26.7%)	17 (35.4%)
≥3	5 (29.4%)	1 (6.2%)	3 (20.0%)	9 (18.8%)

**Table 2:** Comparison of cytokine levels of intra-abdominal ascites fluid of the gastrointestinal cancer subgroups and the comparison group.

	Comparison Group (n:15)	Gastrointestinal Cancer (n:48)			*p* Value ^1^	*p* Value ^2^
		Colorectal Cancer (n:17)	Gastric Cancer (n:16)	Pancreas Cancer (n:15)		
IL1 Beta (pg/mL) [median, (min–max)]	3.21 (1.12–5.01)	2.81 (1.15–16.13)	2.10 (1.04–7.32)	1.93 (1.05–30.38)	0.699	0.785
IFN-Alfa 2 (pg/mL) [median, (min–max)]	0.14 (0.03–0.50)	0.28 (0.04–0.74)	0.17 (0.01–1.78)	0.19 (0.02–1.01)	0.258	0.375
IFN- Gama (pg/mL) [median, (min–max)]	1.71 (0.62–4.58)	2.87 (0.89–9.96)	1.74 (0.98–4.92)	2.09 (0.57–8.55)	0.186	0.156
TNF-Alfa (pg/mL) [median, (min–max)]	2.00 (1.34–4.87)	5.12 (1.22–13.06)	2.75 (1.17–23.25)	3.23 (1.24–27.94)	0.010	0.027
MCP-1 (pg/mL) [median, (min–max)]	1317.74 (379.76–2652.29)	883.88 (166.74–6082.03)	965.47 (388.08–4818.46)	1051.12 (123.12–3226.88)	0.534	0.899
IL-6 (ng/mL) [median, (min–max)]	4.02 (0.01–9.81)	10.60 (5.40–22.43)	11.20 (0.34–29.50)	10.98 (2.70–26.23)	<0.001	0.001
IL-8 (ng/mL) [median, (min–max)]	1.08 (0.01–2.59)	0.95 (0.24–10.37)	5.46 (0.6–20.50)	1.27 (0.44–12.29)	0.146	0.018
IL-10 (pg/mL) [median, (min–max)]	62.78 (14.93–157.61)	113.10 (50.00–583.49)	111.33 (23.43–278.12)	101.67 (15.44–531.05)	0.002	0.010
IL-12p70 (pg/mL) [median, (min–max)]	1.50 (0.59–3.46)	2.58 (1.34–5.70)	2.32 (0.85–5.44)	2.12 (0.43–4.76)	0.001	0.005
IL-17A (pg/mL) [median, (min–max)]	0.09 (0.03–0.27)	0.09 (0.03–0.23)	0.08 (0.02–0.75)	0.12 (0.03–0.62)	0.577	0.779
IL-18 (pg/mL) [median, (min–max)]	234.78 (90.36–542.90)	373.34 (162.18–1078.59)	310.67 (109.54–875.00)	386.57 (75.99–1164.24)	0.013	0.099
IL-23 (pg/mL) [median, (min–max)]	1.84 (1.15–5.37)	4.26 (1.79–9.76)	3.89 (0.77–16.57)	3.74 (0.86–10.72)	0.001	0.005
IL-33 (pg/mL) [median, (min–max)]	15.82 (5.50–33.57)	12.39 (5.65–27.03)	9.98 (5.20–22.80)	9.24 (4.97–47.03)	0.149	0.356

*p value ^1^: Mann–Whitney U for the comparison of malignant ascites (pooled) and benign ascites, p value ^2^: Kruskal–Wallis test for the comparison of disease-specific subsets and benign ascites.*

**Table 3:** Comparison of immune checkpoint levels of intra-abdominal ascites fluid of the gastrointestinal cancer subgroups and the comparison group.

		Gastrointestinal Cancer (n:48)				
	Comparison Group (n:15)	Colorectal Cancer (n:17)	Gastric Cancer (n:16)	Pancreas Cancer (n:15)	*p* Value ^1^	*p* Value ^2^
IL-2R Alpha (sCD25) (ng/mL) [median (min–max)]	2.02 (0.42–10.67)	3.05 (1.08–11.71)	1.65 (0.72–3.80)	2.07 (0.87–4.78)	0.878	0.534
s4-1 BB (sCD137) (pg/mL) [median (min–max)]	6.53 (3.80–12.49)	10.99 (4.40–26.59)	21.10 (4.78–60.72)	9.40 (5.82–21.85)	0.001	0.003
sB7-2 (CD86) (pg/mL) [median (min–max)]	88.97 (20.40–210.78)	103.53 (62.91–288.08)	112.94 (20.66–225.81)	115.02 (52.90–275.67)	0.093	0.350
TGF-Beta 1 (pg/mL) [median (min–max)]	0.60 (0.01–1.44)	0.91 (0.38–2.28)	1.91 (0.46–5.30)	1.71 (0.12–3.99)	0.001	0.001
sCTLA-4 (pg/mL) [median (min–max)]	0.36 (0.02–1.70)	0.40 (0.06–1.60)	0.45 (0.05–2.07)	0.52 (0.20–2.35)	0.321	0.286
sPD-L1 (pg/mL) [median (min–max)]	13.23 (5.03–34.30)	32.91 (11.12–80.49)	17.64 (3.95–38.23)	15.46 (4.48–41.64)	0.108	0.005
sPD-1 (pg/mL) [median (min–max)]	2.99 (1.27–10.87)	3.20 (0.22–8.95)	1.87 (0.15–5.01)	2.99 (1.43–11.34)	0.453	0.059
sTim-3 (ng/mL) (mean ± SD)	65.05 ± 37.60	58.42 ± 38.84	41.69 ± 21.87	46.89 ± 29.54	0.109	0.190
sLAG-3 (pg/mL) [median (min–max)]	1104.85 (162.69–2265.64)	656.95 (133.69–2409.70)	833.90 (133.69–1992.51)	746.62 (109.27–2159.03)	0.150	0.537
sGalectin-9 (ng/mL) [median, (min–max)]	149.13 (46.25–196.62)	173.29 (45.26–363.07)	168.98 (46.00–286.41)	135.26 (66.84–363.07)	0.191	0.610

*p value* *^1^: Student T test for Tim3, Mann–Whitney U test for the other parameters for the comparison of malignant ascites (pooled) and benign ascites, p value ^2^: One-Way ANOVA test for Tim3, Kruskal–Wallis test for the other parameters for the comparison of disease-specific subsets and benign ascites.*

**Table 4:** The results of univariate and multivariate Cox Regression Analysis of the variables according to survival in all cancer patients (HR: Hazard Ratio).

	Univariate Analysis			Multivariate Analysis		
	HR	95% GI	*p* Value	HR	95% CI	*p* Value
IL-10	1.005	1.003–1.008	0.000	1.007	1.002–1.012	0.007
TNF-Alpha	1.103	1.055–1.154	0.000	0.928	0.846–1.117	0.110
IL-6	1.072	1.027–1.118	0.001	1.038	0.970–1.110	0.283
IL-12p70	1.072	0.852–1.350	0.552	N/A	N/A	N/A
IL-18	1.001	0.999–1.002	0.318	N/A	N/A	N/A
IL-23	1.003	0.916–1.098	0.947	N/A	N/A	N/A
s4-1 BB	1.025	1.003–1.048	0.025	1.034	1.002–1.067	0.036
TGF-Beta 1	1.451	1.113–1.892	0.006	1.131	0.808–1.584	0.472
PD-L1	1.011	0.996–1.026	0.140	N/A	N/A	N/A

## Data Availability

The data presented in this study are available on request from the corresponding author due to restrictions protecting patient privacy and the sensitive nature of the clinical data.

## Supplementary Materials

The following supporting information can be downloaded at: https://www.mdpi.com/article/10.3390/biomedicines14051141/s1, Figure S1: Kaplan–Meier curves of overall survival by cancer subtype. (Kaplan–Meier estimates for colorectal (blue), gastric (red), and pancreatic (green) cancer in the pooled malignant cohort (colorectal n = 17, gastric n = 16, pancreatic n = 15). Time-to-event is measured in days from enrollment/ascites sampling to death (event = 1); controls were excluded, and no censoring occurred. Group differences were evaluated with the log-rank (Mantel–Cox) test. Median survival values for each subgroup are reported in the Results; the pancreatic group shows the shortest survival; Figure S2: Correlations between biomarkers and overall survival according to cancer subtype. (Heatmap depicting Pearson correlation coefficient between each biomarker and overall survival (days) in the gastrointestinal malignancies according to cancer subtype (colorectal n = 17, gastric n = 16, pancreatic n = 15). Controls were excluded from survival correlations. Each tile shows r (correlation coefficient); color encodes effect direction and magnitude (stronger color = larger |r|). Time-to-event was defined from study enrollment (ascites sampling) to death; all malignant cases experienced the event (death = 1); Table S1: The comparison of demographic and complete blood count results of all gastrointestinal cancer patients and control volunteers (WBC: White blood cell, ALT: Alanine aminotransferase, AST: Aspartate aminotransferase, SD: Standard derivation); Table S2: The comparison of demographic and complete blood count results of the blood of gastrointestinal cancer subgroups and control volunteers (WBC: White blood cell, ALT: Alanine aminotransferase, AST: Aspartate aminotransferase, SD: Standard derivation); Table S3: Spearman rank correlation coefficients (ρ) between systemic clinical parameters (albumin, AST, direct bilirubin, platelet count) and ascites immune mediators in cancer patients, stratified by cancer subtype.

## References

[1] Nakano M., Ito M., Tanaka R., Yamaguchi K., Ariyama H., Mitsugi K., Yoshihiro T., Ohmura H., Tsuruta N., Hanamura F. (2018). PD-1+ TIM-3+ T cells in malignant ascites predict prognosis of gastrointestinal cancer. Cancer Sci..

[2] Parsons S.L., Lang M.W., Steele R.J. (1996). Malignant ascites: A 2-year review from a teaching hospital. Eur. J. Surg. Oncol..

[3] Fuca G., Cohen R., Lonardi S., Shitara K., Elez M.E., Fakih M., Chao J., Klempner S.J., Emmett M., Jayachandran P. (2022). Ascites and resistance to immune checkpoint inhibition in dMMR/MSI-H metastatic colorectal and gastric cancers. J. Immunother. Cancer.

[4] Wada J., Suzuki H., Fuchino R., Yamasaki A., Nagai S., Yanai K., Koga K., Nakamura M., Tanaka M., Morisaki T. (2009). The contribution of vascular endothelial growth factor to the induction of regulatory T-cells in malignant effusions. Anticancer Res..

[5] Park H.S., Kwon W.S., Park S., Jo E., Lim S.J., Lee C.-K., Lee J.B., Jung M., Kim H.S., Beom S.-H. (2019). Comprehensive immune profiling and immune-monitoring using body fluid of patients with metastatic gastric cancer. J. Immunother. Cancer.

[6] Bamias A., Tsiatas M.L., Kafantari E., Liakou C., Rodolakis A., Voulgaris Z., Vlahos G., Papageorgiou T., Tsitsilonis O., Bamia C. (2007). Significant differences of lymphocytes isolated from ascites of patients with ovarian cancer compared to blood and tumor lymphocytes. Association of CD3+CD56+ cells with platinum resistance. Gynecol. Oncol..

[7] Imai Y., Hasegawa K., Matsushita H., Fujieda N., Sato S., Miyagi E., Kakimi K., Fujiwara K. (2018). Expression of multiple immune checkpoint molecules on T cells in malignant ascites from epithelial ovarian carcinoma. Oncol. Lett..

[8] Naimi A., Mohammed R.N., Raji A., Chupradit S., Yumashev A.V., Suksatan W., Shalaby M.N., Thangavelu L., Kamrava S., Shomali N. (2022). Tumor immunotherapies by immune checkpoint inhibitors (ICIs); the pros and cons. Cell Commun. Signal..

[9] Ari A., Sevik H., Sevinc M.M., Tatar C., Buyukasik K., Surel A.A., Idiz U.O. (2024). Predicting the Response to Neoadjuvant Chemoradiotherapy in Locally Advanced Rectal Cancer Using Soluble Immune Checkpoints. Cancer Biother. Radiopharm..

[10] Marin-Acevedo J.A., Dholaria B., Soyano A.E., Knutson K.L., Chumsri S., Lou Y. (2018). Next generation of immune checkpoint therapy in cancer: New developments and challenges. J. Hematol. Oncol..

[11] Świderska J., Kozłowski M., Nowak K., Rychlicka M., Branecka-Woźniak D., Kwiatkowski S., Pius-Sadowska E., Machaliński B., Cymbaluk-Płoska A. (2022). Clinical Relevance of Soluble Forms of Immune Checkpoint Molecules sPD-1, sPD-L1, and sCTLA-4 in the Diagnosis and Prognosis of Ovarian Cancer. Diagnostics.

[12] Runyon B.A. (1994). Care of patients with ascites. N. Engl. J. Med..

[13] Czajka-Francuz P., Cisoń-Jurek S., Czajka A., Kozaczka M., Wojnar J., Chudek J., Francuz T. (2021). Systemic Interleukins’ Profile in Early and Advanced Colorectal Cancer. Int. J. Mol. Sci..

[14] Zheng J., Wang X., Yu J., Zhan Z., Guo Z. (2022). IL-6, TNF-α and IL-12p70 levels in patients with colorectal cancer and their predictive value in anti-vascular therapy. Front. Oncol..

[15] Lee K.E., Khoi P.N., Xia Y., Park J.S., Joo Y.E., Kim K.K., Choi S.Y., Jung Y.D. (2013). *Helicobacter pylori* and interleukin-8 in gastric cancer. World J. Gastroenterol..

[16] Yan G., Liu T., Yin L., Kang Z., Wang L. (2018). Levels of peripheral Th17 cells and serum Th17-related cytokines in patients with colorectal cancer: A meta-analysis. Cell. Mol. Biol..

[17] Stanilov N., Miteva L., Deliysky T., Jovchev J., Stanilova S. (2010). Advanced Colorectal Cancer Is Associated with Enhanced IL-23 and IL-10 Serum Levels. Lab. Med..

[18] Gilmour B.C., Corthay A., Øynebråten I. (2024). High production of IL-12 by human dendritic cells stimulated with combinations of pattern-recognition receptor agonists. npj Vaccines.

[19] Qi Q., Peng Y., Zhu M., Zhang Y., Bao Y., Zhang X., Zhang J., Liu Y. (2023). Association between serum levels of 12 different cytokines and short-term efficacy of anti-PD-1 monoclonal antibody combined with chemotherapy in advanced gastric cancer. Int. Immunopharmacol..

[20] Czajka-Francuz P., Francuz T., Cisoń-Jurek S., Czajka A., Fajkis M., Szymczak B., Kozaczka M., Malinowski K.P., Zasada W., Wojnar J. (2020). Serum cytokine profile as a potential prognostic tool in colorectal cancer patients—One center study. Rep. Pr. Oncol. Radiother..

[21] Huang Y., Zou K., Jiang H., Li Z. (2024). The complex role of IL-10 in malignant ascites: A review. Cancer Immunol. Immunother..

[22] Neuzillet C., Tijeras-Raballand A., Cohen R., Cros J., Faivre S., Raymond E., de Gramont A. (2015). Targeting the TGFβ pathway for cancer therapy. Pharmacol. Ther..

[23] Pan S., Zhao W., Li Y., Ying Z., Luo Y., Wang Q., Li X., Lu W., Dong X., Wu Y. (2023). Prediction of risk and overall survival of pancreatic cancer from blood soluble immune checkpoint-related proteins. Front. Immunol..

[24] Pitts S.C., Schlom J., Donahue R.N. (2024). Soluble immune checkpoints: Implications for cancer prognosis and response to immune checkpoint therapy and conventional therapies. J. Exp. Clin. Cancer Res..

[25] Bolandi N., Derakhshani A., Hemmat N., Baghbanzadeh A., Asadzadeh Z., Nour M.A., Brunetti O., Bernardini R., Silvestris N., Baradaran B. (2021). The Positive and Negative Immunoregulatory Role of B7 Family: Promising Novel Targets in Gastric Cancer Treatment. Int. J. Mol. Sci..

[26] Pawłowska A., Kwiatkowska A., Suszczyk D., Chudzik A., Tarkowski R., Barczyński B., Kotarski J., Wertel I. (2021). Clinical and Prognostic Value of Antigen-Presenting Cells with PD-L1/PD-L2 Expression in Ovarian Cancer Patients. Int. J. Mol. Sci..

[27] Michel J., Langstein J., Hofstadter F., Schwarz H. (1998). A soluble form of CD137 (ILA/4-1BB), a member of the TNF receptor family, is released by activated lymphocytes and is detectable in sera of patients with rheumatoid arthritis. Eur. J. Immunol..

[28] Kachapati K., Bednar K.J., Adams D.E., Wu Y., Mittler R.S., Jordan M.B., Hinerman J.M., Herr A.B., Ridgway W.M. (2013). Recombinant soluble CD137 prevents type one diabetes in nonobese diabetic mice. J. Autoimmun..

[29] Peng Y., Zhang C., Rui Z., Tang W., Xu Y., Tao X., Zhao Q., Tong X. (2022). A comprehensive profiling of soluble immune checkpoints from the sera of patients with non-small cell lung cancer. J. Clin. Lab. Anal..

[30] Lima C.A.C., Martins M.R., dos Santos R.L., da Silva L.M., Da Silva J.P.A., Forones N.M., Torres L.C. (2024). Soluble levels of 4-1BB (CD137) and OX40 (CD134) are associated with cancer progression in gastric adenocarcinoma. J. Surg. Oncol..

[31] Satala C.B., Bara T.J., Jung I., Tudorache V., Gurzu S. (2021). Chylous Ascites, Unusual Association with Ductal Pancreatic Adenocarcinoma with Plasmacytoid Morphology: A Case Report and Literature Review. Surg. J..

[32] Han M.Y., Borazanci E.H. (2023). Malignant ascites in pancreatic cancer: Pathophysiology, diagnosis, molecular characterization, and therapeutic strategies. Front. Oncol..

[33] Engelmann C., Clària J., Szabo G., Bosch J., Bernardi M. (2021). Pathophysiology of decompensated cirrhosis: Portal hypertension, circulatory dysfunction, inflammation, metabolism and mitochondrial dysfunction. J. Hepatol..

[34] Labelle M., Begum S., Hynes R.O. (2011). Direct signaling between platelets and cancer cells induces an epithelial-mesenchymal-like transition and promotes metastasis. Cancer Cell.

