# Supplementary material for: Immunological Insights into Peritoneal Carcinomatosis for Gastrointestinal Malignancies: The Role of Soluble Factors in Malignant Ascites
Source: Biomedicines. 2026 May 18;14(5):1141. doi: 10.3390/biomedicines14051141 (PMC13205022; doi:10.3390/biomedicines14051141)
Supplement: Supplementary file 1 [file biomedicines-14-01141-s001.zip › Supplementary Tables.pdf]

Supplementary Table S1: The comparison of demographic and complete blood count results of all gastrointestinal cancer patients and control volunteers (WBC: White blood cell, ALT: Alanine aminotransferase, AST: Aspartate aminotransferase, SD: Standard derivation).

| | | Control (n:15) | Gastrintestinale Cancer (n:48) | p value |
|---|---|---|---|---|
| Age (year) (mean±SD) | | 62.06±13.50 | 59.08±14.46 | 0.482 |
| Gender | Female (n,%) | 7 (46.7%) | 16 (33.3%) | 0.349 |
| | Male (n,%) | 8 (53.3%) | 32 (66.7%) | |
| Hematocrit % (mean±SD) | | 31.57±8.29 | 31.06±6.97 | 0.815 |
| WBC ($10^3$/µl) (mean±SD) | | 7.41±2.55 | 9.61±4.26 | 0.064 |
| Neutrophil ($10^3$/µl) [median, (min-max)] | | 4.70 (0.96-11.25) | 4.26 (0.04-16.47) | **0.024** |
| Lymphocyte ($10^3$/µl) [median, (min-max)] | | 1.08 (0.29-2.98) | 1.04 (0.23-4.27) | 0.556 |
| Platelet ($10^3$/µl) (mean±SD) | | 206.40±98.85 | 229.71±132.93 | 0.534 |
| Glucose [median, (min-max)] | | 113.00 (54.00-199.00) | 106.00 (46.00- 232.00) | 0.936 |
| Albumin (mean±SD) | | 34.08±6.14 | 28.02±8.04 | **0.009** |
| Urea [median, (min-max)] | | 61.00 (17.00-187.00) | 44.90 (18.40- 259.00) | 0.498 |
| Creatinine [median, (min-max)] | | 1.05 (0.46-2.20) | 0.82 (0.25-6.40) | 0.809 |
| ALT [median, (min-max)] | | 15.00 (6.00-38.00) | 27.00 (4.00- 737.00) | **0.015** |
| AST [median, (min-max)] | | 22.00 (12.00-83.00) | 47.50 (5.00-1698.00) | **0.021** |
| Sodium (mean±SD) | | 137.75±3.02 | 136.50±6.36 | 0.468 |
| Potassium (mean±SD) | | 4.56±0.78 | 4.50±0.95 | 0.822 |
| Calcium (mean±SD) | | 8.83±0.88 | 8.42±0.95 | 0.142 |
| Bilirubin Direct [median, (min-max)] | | 0.40 (0.05-0.85) | 0 .50 (0.06-18.30) | 0.144 |
| Bilirubin Total [median, (min-max)] | | 1.00 (0.25-1.79) | 1.10 (0.30- 23.10) | 0.550 |
| INR [median, (min-max)] | | 1.25 (0.90- 1.90) | 1.20 (.80-3.50) | 0.764 |

Supplementary Table S2: The comparison of demographic and complete blood count results of the blood of gastrointestinal cancer subgroups and control volunteers (WBC: White blood cell, ALT: Alanine aminotransferase, AST: Aspartate aminotransferase, SD: Standard derivation)

| | | Control (n:15) | Colorectal Cancer (n:17) | Gastric Cancer (n:16) | Pancreas Cancer (n:15) | p value |
|---|---|---|---|---|---|---|
| Age (year) (mean±SD) | | 63.07±13.51 | 61.00±12.65 | 53.19±16.40 | 63.20±12.99 | 0.184 |
| Gender | Female (n,%) | 7 (46.7%) | 5 (29.4%) | 5 (31.3%) | 6 (40.0%) | 0.728 |
| | Male (n,%) | 8 (53.3%) | 12 (70.6%) | 11 (68.8%) | 9 (60.0%) | |
| Hematocrit (%) (mean±SD) | | 31.57±8.29 | 31.47±7.67 | 32.26±5.50 | 29.31±7.65 | 0.709 |
| WBC ($10^3$/µl) (mean±SD) | | 7.41±2.55 | 10.43±3.62 | 10.02±4.72 | 8.25±4.35 | 0.108 |
| Neutrophil ($10^3$/µl) [median, (min-max)] | | 4.70 (0.96-11.25) | 8.33 (2.64-16.45) | 7.27 (0.04-13.62) | 4.90 (0.65-16.47) | 0.082 |
| Lymphocyte ($10^3$/µl) [median, (min-max)] | | 1.08 (0.29-2.98) | 0.89 (0.23-4.27) | 1.21 (0.33-2.67) | 1.02 (0.32-4.10) | 0.628 |
| Platelet ($10^3$/µl) (mean±SD) | | 206.40±98.85 | 209.88±111.14 | 310.13±153.08 | 166.40±89.09 | **0.008** |
| Glucose [median, (min-max)] | | 113.00 (54.00-199.00) | 128.00 (85.00-213.00) | 104.00 (84.00-174.00) | 97.00 (46.00-232.10) | 0.422 |
| Albumin (mean±SD) | | 34.08±6.14 | 28.12±5.81 | 29.42±9.37 | 26.40±8.90 | 0.050 |
| Urea [median, (min-max)] | | 61.00 (17.00-187.00) | 31.90 (18.40-222.00) | 51.05 (22.00-259.00) | 58.60 (29.70-219.30) | 0.294 |
| Creatinine [median, (min-max)] | | 1.05 (0.46-2.20) | 0.75 (0.34-2.67) | 0.82 (0.25-4.80) | 1.30 (0.39-6.40) | 0.457 |
| ALT [median, (min-max)] | | 15.00 (6.00-38.00) | 22.00 (8.00-442.20) | 28.00 (4.00-414.00) | 28.00 (8.00-737.00) | 0.111 |
| AST [median, (min-max)] | | 22.00 (12.00-83.00) | 48.00 (18.00-966.00) | 24.50 (5.00-1438.00) | 49.00 (14.90-1698.00) | **0.029** |

| | | | | | |
|---|---|---|---|---|---|
| Sodium (mean±SD) | 137.75±3.02 | 135.47±7.61 | 137.88±5.58 | 136.20±5.71 | 0.574 |
| Potassium (mean±SD) | 4.56±0.78 | 4.48±1.22 | 4.36±0.69 | 4.68±0.89 | 0.805 |
| Calcium (mean±SD) | 8.83±0.88 | 8.27±0.73 | 8.70±0.78 | 8.28±1.28 | 0.231 |
| Bilirubin Direct [median, (min-max)] | 0.40 (0.05-0.85) | 0.50 (0.10-2.09) | 0.30 (0.06-3.70) | 1.10 (0.40-18.30) | **0.001** |
| Bilirubin Total [median, (min-max)] | 1.00 (0.25-1.79) | 1.23 (0.32-3.65) | 0.81 (0.30-3.90) | 1.70 (1.00-23.10) | **0.002** |
| INR [median, (min-max)] | 1.25 (0.90-1.90) | 1.20 (0.90-2.10) | 1.00 (0.80-3.50) | 1.30 (1.00-2.10) | 0.081 |

**Supplementary Table S3:** Spearman rank correlation coefficients (ρ) between systemic clinical parameters and ascites immune mediators in cancer patients

| **All Cancer (n=48)** | | | | | | | | | |
|---|---|---|---|---|---|---|---|---|---|
| | **TNF-α** | **IL-6** | **IL-8** | **IL-10** | **IL-12p70** | **IL-23** | **s4-1BB** | **TGF-β1** | **sPD-L1** |
| **Albumin** | -0.213 (p=0.147) | -0.211 (p=0.150) | -0.018 (p=0.904) | **-0.307 (p=0.034)*** | 0.226 (p=0.122) | -0.017 (p=0.911) | -0.041 (p=0.781) | -0.255 (p=0.081) | -0.207 (p=0.158) |
| **AST** | -0.032 (p=0.830) | -0.191 (p=0.194) | -0.099 (p=0.505) | -0.041 (p=0.780) | 0.004 (p=0.978) | -0.043 (p=0.773) | -0.095 (p=0.521) | 0.009 (p=0.949) | -0.175 (p=0.235) |
| **Direct Bilirubin** | 0.261 (p=0.074) | 0.065 (p=0.662) | 0.135 (p=0.360) | 0.148 (p=0.316) | -0.064 (p=0.665) | -0.001 (p=0.994) | 0.052 (p=0.728) | 0.087 (p=0.557) | 0.089 (p=0.547) |
| **Platelet** | -0.074 (p=0.618) | -0.120 (p=0.417) | 0.197 (p=0.180) | -0.178 (p=0.227) | 0.019 (p=0.897) | 0.040 (p=0.787) | 0.115 (p=0.435) | 0.041 (p=0.783) | -0.084 (p=0.572) |
| **Colorectal (n=17)** | | | | | | | | | |
| | **TNF-α** | **IL-6** | **IL-8** | **IL-10** | **IL-12p70** | **IL-23** | **s4-1BB** | **TGF-β1** | **sPD-L1** |
| **Albumin** | 0.009 (p=0.974) | -0.234 (p=0.365) | -0.109 (p=0.677) | -0.141 (p=0.589) | 0.234 (p=0.366) | -0.313 (p=0.221) | -0.098 (p=0.708) | -0.159 (p=0.542) | **-0.519 (p=0.033)*** |
| **AST** | -0.291 (p=0.257) | -0.420 (p=0.093) | -0.086 (p=0.743) | -0.260 (p=0.313) | 0.192 (p=0.461) | -0.338 (p=0.184) | -0.283 (p=0.272) | -0.284 (p=0.269) | -0.388 (p=0.124) |
| **Direct Bilirubin** | 0.233 (p=0.368) | 0.357 (p=0.160) | **0.487 (p=0.047)*** | 0.389 (p=0.123) | 0.329 (p=0.197) | 0.145 (p=0.579) | 0.189 (p=0.468) | 0.002 (p=0.994) | 0.229 (p=0.376) |
| **Platelet** | -0.204 (p=0.433) | -0.366 (p=0.149) | -0.129 (p=0.622) | -0.250 (p=0.333) | **-0.497 (p=0.043)*** | -0.366 (p=0.149) | -0.005 (p=0.985) | -0.009 (p=0.974) | -0.457 (p=0.065) |
| **Gastric (n=16)** | | | | | | | | | |
| | **TNF-α** | **IL-6** | **IL-8** | **IL-10** | **IL-12p70** | **IL-23** | **s4-1BB** | **TGF-β1** | **sPD-L1** |
| **Albumin** | -0.213 (p=0.427) | -0.007 (p=0.978) | -0.143 (p=0.598) | -0.246 (p=0.359) | 0.375 (p=0.152) | 0.278 (p=0.297) | 0.069 (p=0.799) | -0.487 (p=0.056) | 0.107 (p=0.694) |
| **AST** | -0.057 (p=0.833) | -0.128 (p=0.636) | 0.040 (p=0.884) | -0.066 (p=0.807) | -0.230 (p=0.392) | -0.119 (p=0.660) | -0.052 (p=0.850) | 0.099 (p=0.716) | -0.309 (p=0.244) |
| **Direct Bilirubin** | 0.083 (p=0.761) | -0.040 (p=0.883) | 0.266 (p=0.320) | 0.001 (p=0.996) | -0.310 (p=0.243) | -0.211 (p=0.433) | 0.165 (p=0.541) | 0.125 (p=0.643) | 0.231 (p=0.390) |

| | | | | | | | | | |
|---|---|---|---|---|---|---|---|---|---|
| **Platelet** | 0.396 (p=0.129) | 0.052 (p=0.850) | 0.230 (p=0.392) | -0.013 (p=0.961) | 0.296 (p=0.266) | 0.390 (p=0.135) | 0.096 (p=0.725) | 0.031 (p=0.910) | 0.067 (p=0.805) |
| **Pancreatic (n=15)** | | | | | | | | | |
| | **TNF-α** | **IL-6** | **IL-8** | **IL-10** | **IL-12p70** | **IL-23** | **s4-1BB** | **TGF-β1** | **sPD-L1** |
| **Albumin** | -0.207 (p=0.459) | -0.296 (p=0.283) | -0.064 (p=0.820) | -0.339 (p=0.216) | 0.182 (p=0.516) | 0.120 (p=0.671) | -0.229 (p=0.413) | -0.268 (p=0.334) | -0.071 (p=0.800) |
| **AST** | -0.120 (p=0.671) | -0.036 (p=0.899) | 0.189 (p=0.499) | -0.048 (p=0.864) | 0.116 (p=0.680) | 0.124 (p=0.659) | -0.011 (p=0.970) | 0.214 (p=0.443) | -0.164 (p=0.558) |
| **Direct Bilirubin** | 0.249 (p=0.372) | 0.242 (p=0.386) | 0.333 (p=0.226) | 0.292 (p=0.292) | 0.224 (p=0.423) | 0.114 (p=0.687) | 0.352 (p=0.198) | 0.501 (p=0.057) | 0.091 (p=0.746) |
| **Platelet** | -0.120 (p=0.671) | -0.311 (p=0.259) | 0.322 (p=0.242) | -0.216 (p=0.439) | 0.066 (p=0.815) | 0.107 (p=0.703) | -0.005 (p=0.985) | -0.189 (p=0.499) | 0.231 (p=0.408) |
