# Supplementary figures and images for: Immunological Insights into Peritoneal Carcinomatosis for Gastrointestinal Malignancies: The Role of Soluble Factors in Malignant Ascites
Source: Biomedicines. 2026 May 18;14(5):1141. doi: 10.3390/biomedicines14051141 (PMC13205022; doi:10.3390/biomedicines14051141)

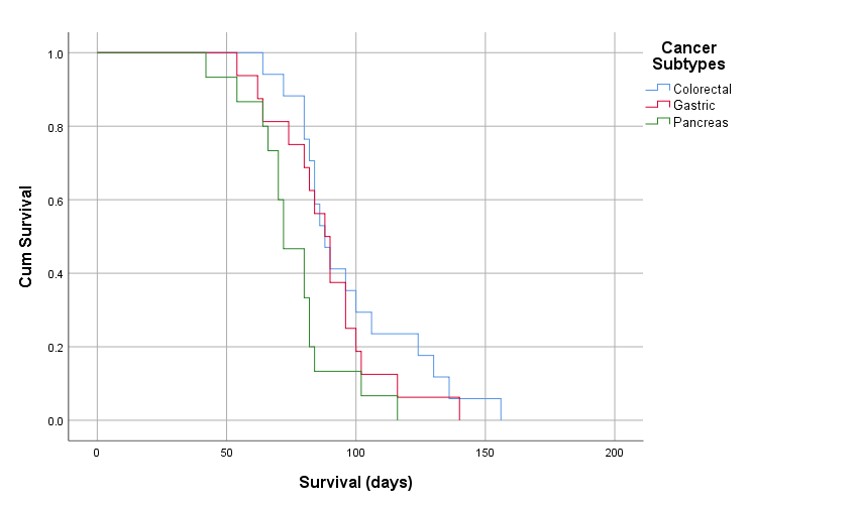

Supplement: Supplementary file 1 [file biomedicines-14-01141-s001.zip › Supplementary Figure S1.jpg]

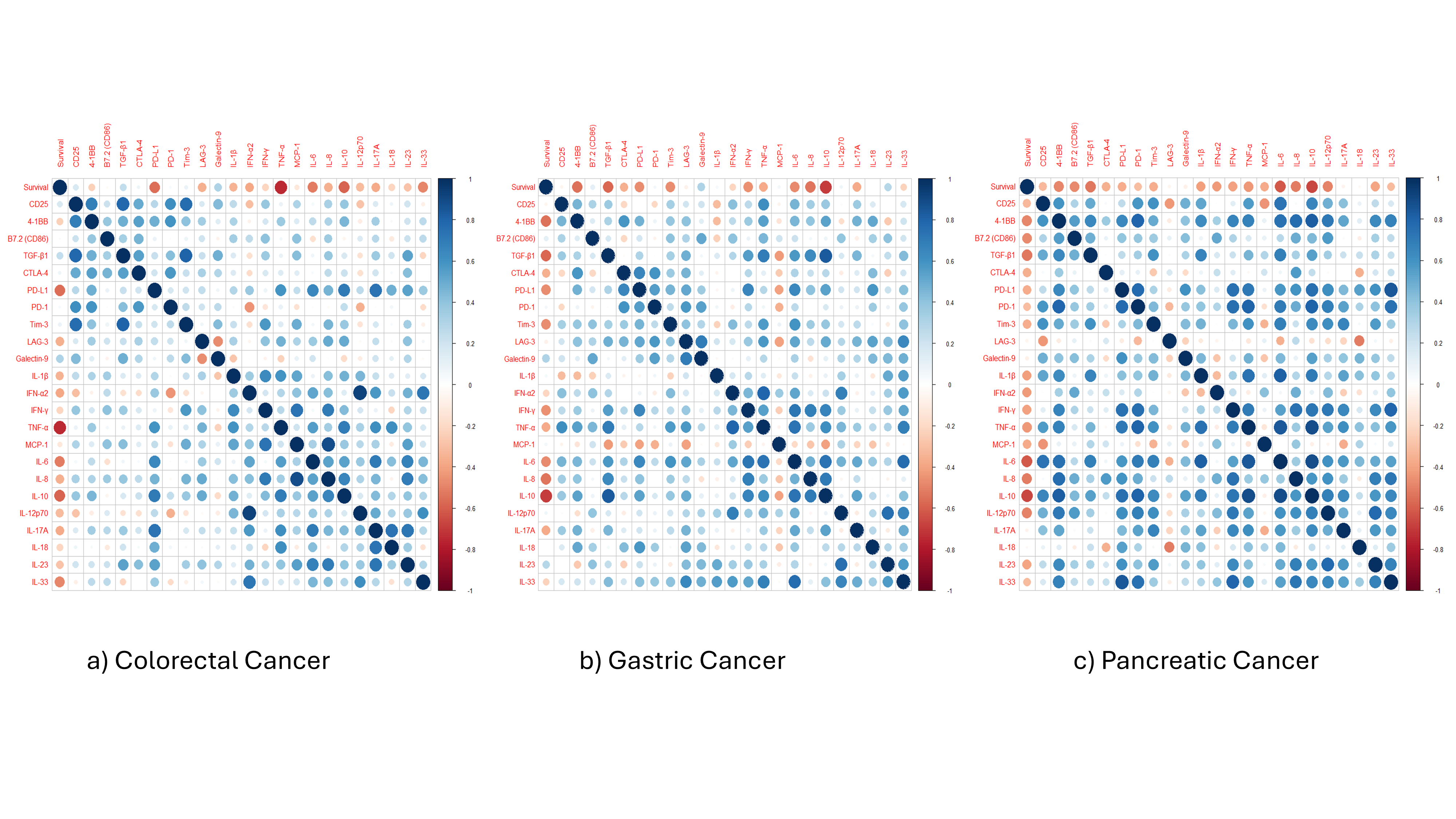

Supplement: Supplementary file 1 [file biomedicines-14-01141-s001.zip › Supplementary Figure S2.tif]
